# Hidden threat of tortoise ticks: high prevalence of Crimean-Congo haemorrhagic fever virus in ticks *Hyalomma aegyptium* in the Middle East

**DOI:** 10.1186/1756-3305-7-101

**Published:** 2014-03-11

**Authors:** Pavel Široký, Tomáš Bělohlávek, Ivo Papoušek, David Jandzik, Peter Mikulíček, Michaela Kubelová, Lenka Zdražilová-Dubská

**Affiliations:** 1 Department of Biology and Wildlife Diseases, Faculty of Veterinary Hygiene and Ecology, University of Veterinary and Pharmaceutical Sciences Brno, Palackého tř. 1/3, 612 42 Brno, Czech Republic; 2 CEITEC-Central European Institute of Technology, University of Veterinary and Pharmaceutical Sciences Brno, Palackého tř. 1/3, 612 42 Brno, Czech Republic; 3 Department of Zoology, Comenius University in Bratislava, Mlynská dolina B-1, 84215 Bratislava, Slovakia; 4 Department of Ecology and Evolutionary Biology (EBIO), University of Colorado, Boulder, Ramaley N122, Campus Box 334, Boulder, CO 80309-0334, USA; 5 Department of Laboratory Medicine, Masaryk Memorial Cancer Institute, Žlutý kopec 7, 65653 Brno, Czech Republic

**Keywords:** Tick-borne disease, Epidemiology, Tortoises, *Testudo graeca*, *Hyalomma*, Syria, Turkey, RT-PCR

## Abstract

It is the first time that Crimean-Congo haemorrhagic fever virus (CCHFV), causing potentially lethal disease of humans, has been reported from the Middle East region and from the tortoise tick *Hyalomma aegyptium* from a tortoise host, whose epidemiological significance may have remained almost completely overlooked so far. We used RT-PCR to screen for 245 ticks collected from 38 *Testudo graeca* tortoise individuals. Results of our genetic screening provide unambiguous evidence of occurrence of CCHFV in this region and host, suggesting a potentially important role of *H. aegyptium* in CCHF epidemiology.

## 

Crimean-Congo haemorrhagic fever (CCHF) is a potentially fatal viral infection of humans with a distributional range covering the largest geographical area of all medically important tick-borne viral zoonoses [1]. Wildlife rodents, hares and hedgehogs are the most common hosts in endemic areas, while migratory birds can spread CCHF virus (CCHFV) over long distances into new regions [2]. Ticks of the genus *Hyalomma*, particularly *H. marginatum*, are considered principal vectors of CCHFV [3]. *Hyalomma* ticks mostly feed on mammals, although pre-adult stages of some species also parasitize on birds. *H. aegyptium*, a three-host tick species, represents a single exception, in which the adults are host-specific, feeding on tortoises of the genus *Testudo*[4]. On the other hand, larvae and nymphs show lower host-specificity and commonly infest various reptiles, small mammals, and birds. Ticks with such a wide host spectrum have greater potential for transmitting pathogenic agents among various vertebrate groups; they can thus act as “bridge vectors” and significantly contribute to spreading its natural foci. Despite this fact, ticks parasitizing reptiles have traditionally been considered epidemiologically and economically less important than ticks of mammals and birds, and as a result, they have been understudied and their vector capability may have been overlooked. However, a relationship between *H. aegyptium* and some important zoonotic agents has recently been suggested [5].

To study the importance of *H. aegyptium* in the epidemiology of CCHF, we took advantage of extensive sampling of this tick species from areas close to a known endemic region of CCHFV occurrence in southern Turkey and a region in north-western Syria (for distribution of sampling sites see Figure 1), which have not been screened for CCHFV so far. In total, we analyzed 245 adult individuals of *H. aegyptium* (Table 1) collected from 38 spur-thighed tortoises (*Testudo graeca*) during the field trips realized in April 2005 and April, May, and June 2007. RNA isolation and RT-PCR amplifying S-segment of CCHFV followed the standard procedures [6], but due to initial difficulties in amplifications and inconsistent results of RT-PCR, we employed the following modifications: decreased total volume of 25 μl for each of the two PCR rounds, reverse transcription for 60 min, and increased annealing temperature at 47°C. In addition, all samples were examined for the presence of CCHFV by repeated RT-PCRs (standard and modified) to eliminate false negative results. In an attempt to identify genotypes and assess the genealogy of our isolates, we sequenced 22 of the obtained PCR products. All newly produced sequences were identical [GenBank® accession nos. KF725870-KF725873]. Their comparison with GenBank® database using the BLAST showed that our sequences differ in a single nucleotide from CCHFV strain IbAr10200 from Nigeria. Bayesian inference carried out using GTR + Γ + I (Generalised time reversible model of substitution evolution, with parameters of gamma distribution and proportion of invariable sites estimated from the data) for 10^7^ generations and maximum likelihood analysis with GTR + Γ + I model also confirmed that our isolates belong to the genotype 3/Africa-3 clade instead of geographically closer European or Asian clades [7,8] (Figure 2).

**Figure 1:**
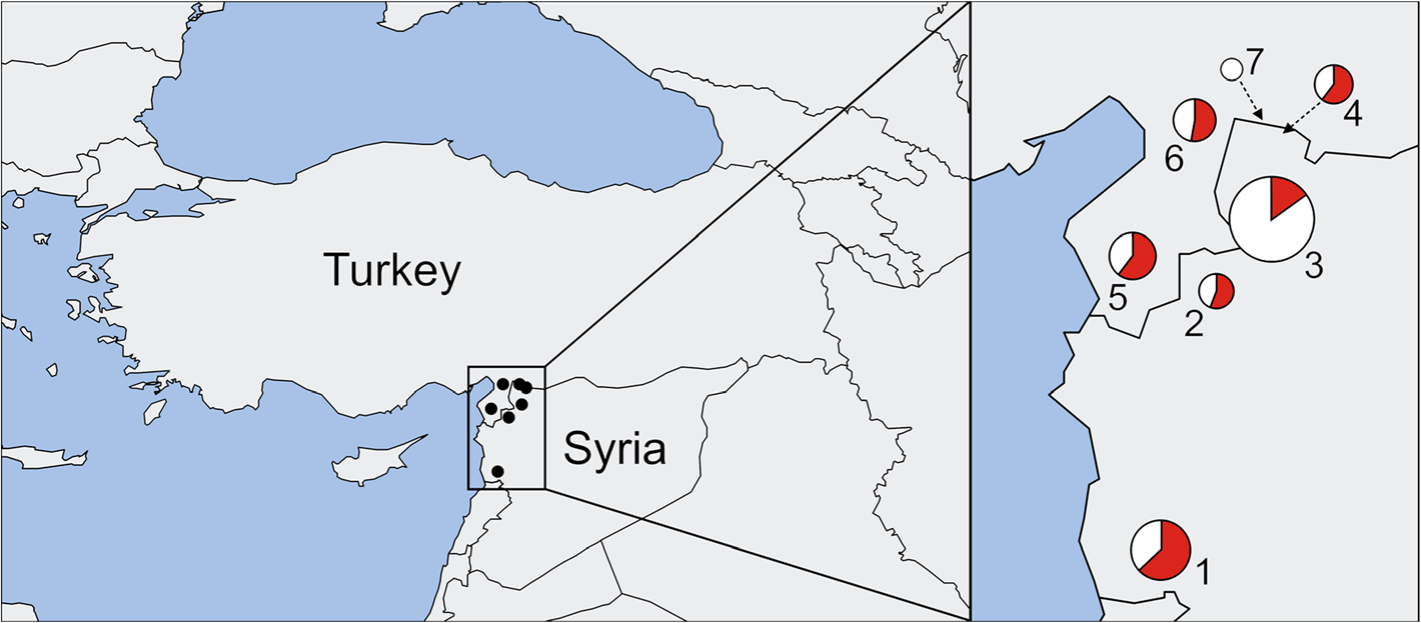
**Map showing sampling localities and relative prevalence of CCHFV – portion of positive samples is shown in red.** The size of each circle corresponds to the sample size from each particular locality. Localities: 1-Krak des Chevaliers, 2-Kafr Takharim, 3-Qalat Samaan, 4-Cirrus, 5-Antakya, 6-Hassa, 7-Bogazkerim.

**Table 1:** **Distribution and prevalence of CCHFV-positive *Hyalomma aegyptium *** **ticks at studied localities in Syria (SY) and Turkey (TR)**

**Locality**	**Coordinates**	**Sampled tortoises/carriers of positive ticks**	**Ticks examined/positive**
Krak des Chevaliers, SY	34°45′N, 36°17′E	7/7	40/25
Kafr Takharim, SY	36°07′N, 36°28′E	2/2	9/5
Qalat Samaan, SY	36°22′N, 36°51′E	19/10	137/20
Cirrus, SY	36°45′N, 36°52′E	2/2	10/4
Antakya, TR	36°12′N, 36°10′E	4/4	25/10
Hassa, TR	36°48′N, 36°29′E	3/2	19/10
Bogazkerim, TR	36°49′N, 36°51′E	1/0	5/0
Total		38/27	245/74

**Figure 2:**
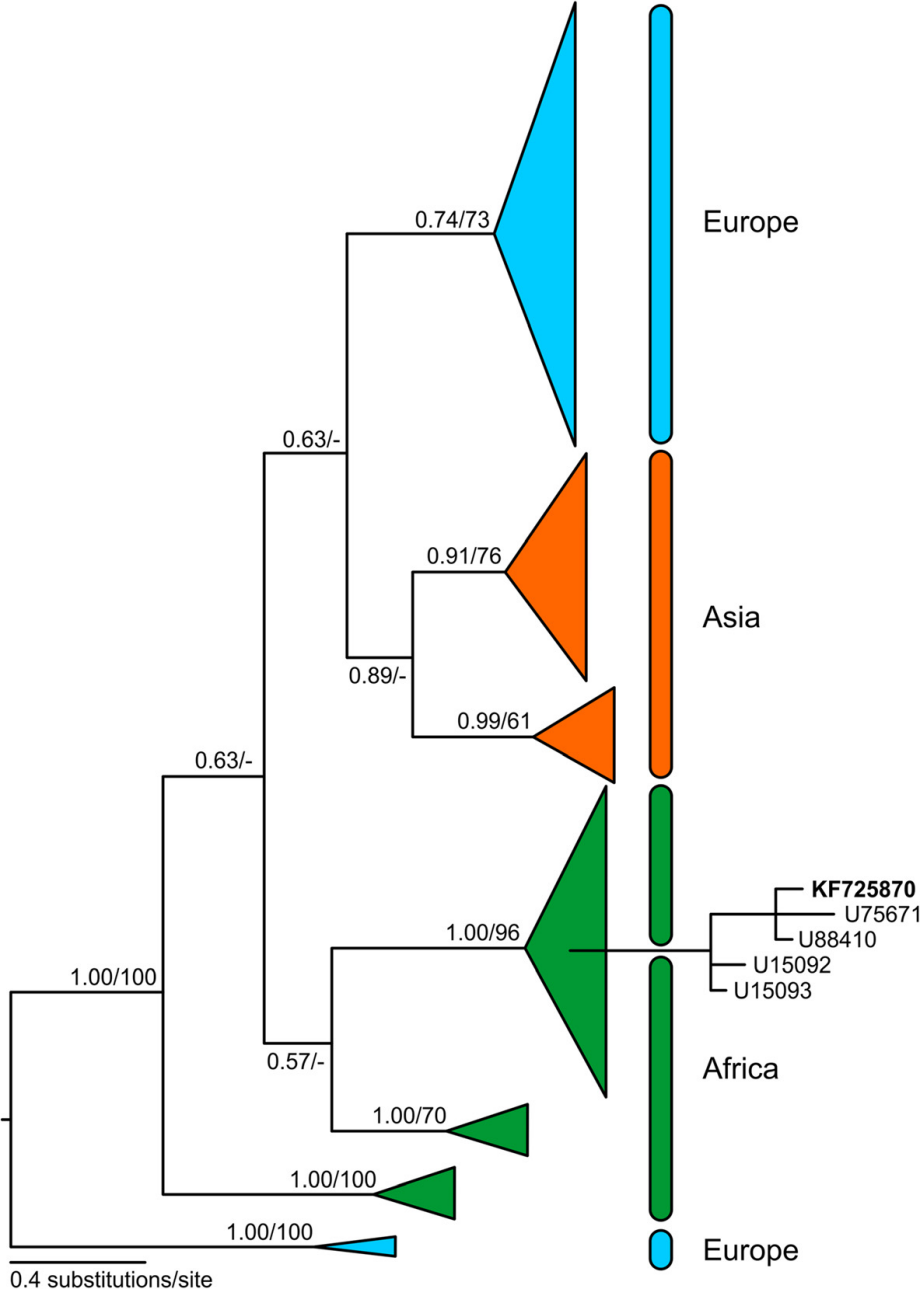
**Bayesian phylogeny of the known haplotypes of CCHFV.** Terminal branches of the main geographically-defined clades were collapsed to ease orientation. Note the paraphyletic nature of the European and African clades as opposed to the monophyletic Asian clade. The heights of collapsed clades are proportional to the number of their haplotypes and the lengths of branches leading to the last common ancestor of each main clade reflect their nucleotide divergence. Detail is given for the sub-clade containing haplotype of our samples from Levant illustrating its clear relationship to African CCHFV haplotypes. The numbers at branches show tree support as Bayesian posterior probabilities/maximum likelihood bootstraps (after 1000 resampled datasets) for each particular branch. Values below 0.5/50 are not shown.

Our results revealed 30.2% (74/245) prevalence of CCHFV in studied *H. aegyptium* (Table 1). Interestingly, the only reptile species detected as a CCHFV antibody carrier so far, was another tortoise species *Testudo horsfieldii*[9], which, similar to *T. graeca*, belongs to *H. aegyptium* hosts. Despite these results, it remains unknown whether tortoises can serve as a reservoir of CCHFV in natural circulation or can only carry infected ticks; to resolve this it would be necessary to confirm a viremic tortoise host. However, since the pre-adult stages of tortoise tick *H. aegyptium* also feed on humans [10], this species can potentially play an important role in CCHF epidemiology, at least in the region of the Middle East.

## Competing interests

The authors declare that they have no competing interests.

## Authors’ contributions

PŠ and LZ-D conceived and designed the study, PŠ, DJ, and PM carried out the field collection of samples, TB and MK carried out the majority of the laboratory work, IP ran phylogenetic analysis, PŠ wrote the manuscript with a little help from DJ who also prepared the figures, all authors read, discussed, and approved the final version of the manuscript.
